# Inherent Serum Inhibition of Influenza Virus Neuraminidases

**DOI:** 10.3389/fvets.2021.677693

**Published:** 2021-08-02

**Authors:** Amanda L. Skarlupka, Ted M. Ross

**Affiliations:** ^1^Center for Vaccines and Immunology, University of Georgia, Athens, GA, United States; ^2^Department of Infectious Diseases, University of Georgia, Athens, GA, United States

**Keywords:** neuraminidase, vaccine, ELLA, influenza, animal

## Abstract

Influenza virus vaccines have been designed for human and veterinary medicine. The development for broadly protective influenza virus vaccines has propelled the vaccine field to investigate and include neuraminidase (NA) components into new vaccine formulations. The antibody-mediated protection induced by NA vaccines is quantified by inhibition of sialic acid cleavage. Non-immune inhibitors against influenza viruses naturally occur in varying proportions in sera from different species. In this brief report, the inherent ability of raw animal sera to inhibit a panel of influenza virus NA was determined. Raw sera from the same species inhibited more than 50% of influenza viruses tested from four different subtypes, but the breadth of inhibiting NA activity depended on the source of sera. Furthermore, different influenza viruses were inhibited by different sources of sera. Overall, additional studies are needed to ensure that scientific methods are consistent across studies in order to compare NA inhibition results. Through future investigation into the differences between sera from different animal species and how they influence NA inhibition assays, there can be effective development of a broadly protective influenza virus vaccines for veterinary and human use.

## Introduction

Influenza viruses are global zoonotic and human pathogens, and vaccination remains the main preventative measure against infection. The influenza virus is a member of the Orthomyxoviridae family. The genome is composed of eight negative single-sense RNA segments that determines the viral genus, alpha-, beta-, delta-, and gammainfluenzavirus that correspond to the species influenza A, B, D, and C viruses, respectively. Of the four influenza types, Types A and D are commonly isolated from animals, whereas influenza Types B and C are most commonly associated with human infection especially in children (1). The Type A influenza viruses are further classified into subtypes determined by the two major surface proteins, hemagglutinin (HA) and neuraminidase (NA). Currently, there are 18 HA subtypes and 11 NA subtypes that can be paired to create different influenza subtypes.

Influenza viruses are of international importance due to the widespread infection in different livestock, leading to vaccination being utilized across the veterinary field (2). Equine influenza viruses are important horse pathogens with policies in place that require horses be vaccinated for equine influenza viruses before participation in events or importation (3, 4). Furthermore, due to the transmission of influenza viruses from horses to dogs, as well as the endemic infection of influenza viruses in the canine population, canine vaccination is also recommended for dogs with high risk of exposure (5, 6). The swine industry uses primarily whole inactivated vaccines [WIVs—reviewed in reference (7)] that are developed using split-inactivated technologies (7, 8). The poultry industry utilizes the greatest variety of vaccine platforms, including split-inactivated virus, HA protein antigens, HA DNA vaccines, and recombinant technologies with other backbone viruses (9, 10).

However, during infection both HA and NA proteins are targets for neutralizing antibodies (11). The NA glycoprotein mediates viral egress and virion de-aggregation by cleaving sialic acids as well as contributing to motility through cleaving mucins in the upper respiratory tract (12, 13). Polyclonal NA-specific sera and NA inhibition (NAI) titers reduce, modulate, and protect against disease (14, 15). Further research has identified monoclonal NA-specific antibodies that neutralize viral growth (16). Although NA antibodies hinder viral replication, the induction of NAI antibody titers following vaccination is not as great as the induction of HAI titers, potentially due to either the split-inactivated vaccines lacking a standardized concentration of NA protein or the NA protein being destroyed during the split-inactivation process (15). Recently, research and vaccine development have focused on live-attenuated viruses that elicit NA antibodies, or protein vaccines that include the NA (17).

Currently, the enzyme-linked lectin assay (ELLA), MUNANA substrate, thiobarbituric acid (TBA) fluorescent-based assay, and NA-Star chemiluminescent assay are methods for measuring antibodies against the NA molecule (18–23). As the NA glycoprotein undergoes antigenic drift, the protein's ability to cleave sialic acid can be measured and quantified using these assays. All techniques assess the elicited antibody-specific inhibition of the NA after vaccination or infection. The ELLA measures the ability of the viral NA to cleave sialic acids from a large substrate (fetuin) similar to infection when sialic acids are expressed on the surface of the host cell, whereas the MUNANA and NA-Star techniques measure cleavage of small soluble chemical substrates (24). However, only the ELLA was proposed as the assay for measuring serum NA-inhibiting antibodies as a correlate for protection for humans (25).

Components in raw sera have non-specific inhibitory activity against NA activity (20). These initial findings were conducted with ferret sera that varied using different viruses from different influenza subtypes. However, treating sera with receptor-destroying enzyme (RDE) overnight and then heat-inactivating the sera for 8 h at 55°C mitigated the non-specific inhibition without loss of NA- or HA-specific inhibitory activity (20). The animal models used for influenza virus research are growing and now include more species. Not only is there a need to compare serological results between animal models that are used for human influenza viruses, but also endemic influenza virus infection in agricultural animal species requires a consistent method to quantify the NA-inhibiting antibodies as well. Therefore, it may be necessary to handle sera from different species differently when quantifying the NA inhibition responses, which may be key to determining overall vaccine effectiveness.

Therefore, animal sera from different species were characterized for their inherent inhibition of the ELLA with a panel of influenza viruses. Sera were compared for their ability to non-specifically inhibit the NA proteins of many influenza viruses representing different viral subtypes. Sera were collected and tested from varying animal serum sources against H1 and H3 human- and swine-isolated influenza strains as well as avian-isolated viruses with N2 and N3 proteins. Overall, there are many different variables that contribute to the interpretation of the ELLA assay, and understanding the innate characteristics of the host origin of the sera is critical to conducting the assay and interpreting the results. Therefore, it is important to standardize methodologies that will allow for consistent and reproducible results to assess anti-NA antibodies.

## Materials and Methods

### Viruses

All swine viruses were passaged once in Madin–Darby canine kidney (MDCK) cell culture at 37°C, which was the same growth conditions as they were received in (26). The harvested virus was centrifuged at 2,500 rpm for 10 min to remove cell debris. Human and avian influenza viruses were propagated in 11-day-old embryonated chicken eggs. Virus lots were aliquoted for single-use applications and stored at −80°C. Viral titer of the frozen aliquots was determined with a plaque assay using MDCK cell culture in plaque-forming units per ml (PFU) (Table 1). The panel of viruses covered a range of N1 to N3 influenza NA subtypes, including A/Brisbane/59/2007 (H1N1) (Bris/07), A/California/07/2009xPR8 (6:2 viral reassortant with six internal genes from A/Puerto Rico/8/1934, and NA and HA external genes from virus indicated) (H1N1) (CA/09), A/swine/Nebraska/A10444614/2013 (H1N1) (Sw/NE/13), A/Vietnam/1203/2004xPR8 (H5N1) (Viet/04; HA gene contains mutation in multibasic cleavage site for BSL-2-level research), A/swine/Missouri/A10444664/2013 (H1N2) (Sw/MO/13), A/swine/North Carolina/152702/2015 (H1N2) (Sw/NC/15), A/white-fronted goose/Netherlands/22/1999 (H2N2) (Wfg/Neth/99), A/quail/Rhode Island/16-018622-1/2016 (H2N2) (Qu/RI/16), A/Port Chalmers/1/1973 (H3N2) (PC/73), A/Hong Kong/4801/2014 (H3N2) (HK/14), A/swine/Missouri/2124514/2006 (H2N3) (Sw/MO/06), and A/mallard/Minnesota/A108-3437/2008 (H2N3) (Mal/MN/08).

**Table 1:** Linear regression fit of the NA activity of the viruses tested in the panel.

				**NA activity reciprocal titer**			
**Strain**	**PFU/ml**	**Fitted equation**	***R*^**2**^**	**100%**	**95%**	**90%**	**ELLA**
Bris/07	4.2 × 10^8^	OD = −0.5795log_2_(Titer) + 7.72	0.9867	160	197	243	200
CA/09	1.9 × 10^8^	OD = −0.5903log_2_(Titer) + 8.804	0.9835	320	402	505	450
Sw/NE/13	1.15 × 10^8^	OD = −0.5694log_2_(Titer) + 7.556	0.9867	160	196	241	200
Viet/04	1.75 × 10^8^	OD = −0.4799log_2_(Titer) + 5.66	0.9738	100	119	143	130
Sw/MO/13	1.31 × 10^7^	OD = −0.5177log_2_(Titer) + 5.932	0.9786	40	49	61	50
Sw/NC/15	8.45 × 10^5^	OD = −0.4144log_2_(Titer) + 3.913	0.9430	10	12	15	15
Wfg/Neth/99	1.0 × 10^8^	OD = −0.6189log_2_(Titer) + 10.86	0.9900	6,400	7,576	8,981	8,000
Qu/RI/16	8.0 × 10^9^	OD = −0.6213log_2_(Titer) + 10.82	0.9896	6,400	7,551	8,911	8,000
PC/73	9.0 × 10^8^	OD = −0.6022log_2_(Titer) + 8.33	0.9789	640	749	875	800
HK/14	3.0 × 10^7^	OD = −0.4438log_2_(Titer) + 3.773	0.9903	10	12	14	13
Sw/MO/06	2.0 × 10^8^	OD = −0.676log_2_(Titer) + 9.549	0.9899	320	390	477	400
Mal/MN/08	4.0 × 10^6^	OD = −0.6394log_2_(Titer) + 11.19	0.9862	3,200	3,911	4,792	4,000

*The plaque-forming units (PFU/ml) and the fitted linear regression equation using a minimum of five two-fold serial dilution data points with the final R-squared value are provided. From the 100% NA activity titer, the 95% and 90% NA activity titers were calculated from the fitted equation. The viral dilution used for the ELLA assay was chosen between that range*.

### Animal Serum

Animal serum was either commercially sourced or generated in house. Sera were confirmed to be negative for preexisting antibodies to currently circulating human influenza viruses by HAI. Ferret sera originated from 6 to 8-months female finch ferrets (*Mustela putorius furo*, spayed, female, 6–8 months, descented) purchased from Triple F Farms (Sayre, PA); porcine sera originated from piglets at Auburn University; and rhesus macaque (*Macaca mulatta*) sera originated from previous dengue virus studies performed in the lab (27). The rat (cat #: 10710C), goat (cat #: 01-6201), horse (cat #: 31874), and mouse (cat #: 01-6501; NIH Swiss mouse) normal sera were harvested from non-immune animals (Invitrogen, Carlsbad, CA, USA) and rehydrated according to the manufacturer's specification; only one lot was tested for each commercial serum. Raw serum was not diluted any further before experimentation.

### NA Activity and Inhibition Assay

High-affinity Immunoblot 4HBX 96-well flat-bottom plates (Thermo Fisher Scientific, Waltham, MA, USA) were coated overnight with 100 μl of 25 μg/ml fetuin (Sigma-Aldrich, St. Louis, MO, USA) in coating buffer (KPL coating solution concentrate; SeraCare Life Sciences Inc., Milford, MA, USA) and stored away from light for a maximum of 2 months at 4°C until use. Viruses were diluted to an initial dilution of 1:10 with Dulbecco's phosphate-buffered saline (DPBS) with Tween-20 and 1% BSA (DPBS-T-B), a PBS which contains 0.133 g/l CaCl_2_ and 0.1 g/l MgCl_2_ further supplemented with 1% BSA, and 0.5% Tween-20. Before virus addition, fetuin plates were washed three times in PBS-T (PBS + 0.05% Tween-20). Virus was diluted in two-fold serial dilutions within a range that allowed for linear regression analysis. After which, 50 μl of the viral dilutions was added to the fetuin-coated plate containing 50 μl of DPBS-T-B in duplicate. A negative control column was included containing 100 μl DPBS-T-B only. Plates were sealed and incubated for 16–18 h at 37°C and 5% CO_2_. After incubation, plates were washed six times in PBS-T. After washing, a diluted lectin was added to the plates to bind exposed galactose. Specifically, 100 μl of peanut agglutinin-HRPO (Sigma-Aldrich, St. Louis, MO, USA) diluted 1,000-fold in DPBS-B (DPBS, 1% BSA). Plates were incubated at RT for 2 h. Plates were washed three times in PBS-T, and 100 μl (500 μg/ml) of o-phenylenediamine dihydrochloride (OPD; Sigma-Aldrich, St. Louis, MO, USA) in 0.05 M phosphate-citrate buffer with 0.03% sodium perborate pH 5.0 (Sigma-Aldrich, St. Louis, MO, USA) was added to the plates. Plates were immediately incubated in the dark for 10 min at room temperature (20−22°C). The reaction was stopped with 100 μl of 1 N sulfuric acid. The absorbance was read at 490 nm. NA activity was determined after subtracting the mean background absorbance of the negative control wells. Linear regression analysis was used to determine the dilution of NA antigen necessary to achieve 90−95% NA activity and was used for subsequent NA inhibition ELLAs.

From each virus titration, at least five serial dilutions within the linear range were used to calculate the linear regression after transforming the dilutions by log_2_. The R-squared value above 0.9 was considered acceptable. The best-fit values for the slope (m) and y-intercept (b) were used to determine the 90−95% range. The lowest titer dilution used for regression was defined as the 100% NA activity dilution. Using the fitted linear regression equation, the optical density (OD_100%_) value for 100% NA activity was calculated. Then, the OD_95%_ and OD_90%_ were calculated by multiplying OD_100%_ by 0.95 and 0.9, respectively. The range of viral dilution for 90−95% NA activity was then determined by using the OD_95%_ and OD_90%_ values in the linear regression equation to obtain lower and upper bounds for the virus dilution (Equation 1). Virus dilutions were then chosen between that range as indicated (Table 1).

(1) $$\begin{array}{l} \;OD=m*\log_{2}\left(Titer\right)+b\\ \;OD_{100\%}=m*\log_{2}\left(Lowest\;Titer\right)+b\\ \;OD_{90\%}=0.9*OD_{100\%}\;OD_{95\%}=0.95*OD_{100\%}\\ Titer_{90\%}=\;2^{\frac{OD_{90\%}-b}{m}}\;Titer_{95\%}=\;2^{\frac{OD_{95\%}-b}{m}} \end{array}$$

The NI ELLA titers were determined from two-fold serially diluting sera in DPBS-T-B from 1:10 to 1:1,280. Duplicate dilutions were added to fetuin plates in 50 μl. The NA antigen was diluted to 90−95% NA activity in DPBS-T-B, and 50 μl was added to the plate. Controls were each a minimum of eight wells and included a positive NA antigen control (50 μl NA antigen + 50 μl DPBS-T-B) and a negative control (100 μl of DPBS-T-B) on each plate. Plates were incubated for 16–18 h at 37°C and 5% CO_2_ after which they were washed and processed, and absorbance was read as described above. Initially, the mean background absorbance from the negative control wells was subtracted from all wells. Then, NA percent activity was determined by dividing the serum absorbance by the mean virus-positive control wells multiplied by 100 (Equation 2).

(2) $$\begin{array}{l} NA\;Activity\;\%\\ \;=\frac{Individual\;Well\;Absorbance}{Mean\;Absorbance\;of\;Virus\;only\;control\;wells}*100 \end{array}$$

Non-linear regression fits were performed using GraphPad Prism version 9.1.1 (223) for MacOS (GraphPad Software, San Diego, CA, USA; www.graphpad.com), and the 50% NAI titer was estimated. Briefly, the “[Agonist] vs. normalized response—Variable slope” model was chosen which fits the model presented in Equation 3, which estimates the Hill slope and the half effective concentration (EC_50_). Outliers were not detected for or removed, and least-square regression with no weighting was used for the fitting. The model was constrained in that EC_50_ was >0. Asymmetrical profile-likelihood 95% confidence intervals of the EC_50_ were determined as well.

(3) $$\begin{array}{r} y=100\frac{x^{Hill\;Slope}}{EC_{50}^{Hill\;Slope}+x^{Hill\;Slope}} \end{array}$$

The lower limit of detection was 1:10, and the upper limit of detection was 1:1,280 due to the range of sera dilution tested.

## Results

### NA Titers of Influenza Viruses

The lowest dilution of virus needed to induce 100% NA activity varied between 1:10 and 1:6,400 for different influenza viruses (Table 1). Three HXN2 viruses had 100% NA titers below 100, 1:40 for Sw/MO/13 (H1N2), and 1:10 for both Sw/NC/15 (H1N2) and HK/14 (H3N2). Of these, the virus titer for only Sw/NC/15 was comparatively low at 8.45 × 10^5^ PFU/ml, while the virus titers for Sw/MO/13 and HK/14 were 2.0 × 10^8^ PFU/ml and 3.0 × 10^7^ PFU/ml, respectively. The avian lineage H2N2 and H2N3 viruses had the highest 100% NA titers of 1:3,200 for Mal/MN/08 (H2N3) and 16,400 for both Wfg/Neth/99 (H2N2) and Qu/RI/16 (H2N2). The virus titer was not greater for these viruses than the others, therefore indicating that the increase in activity is not due to solely an increase in replicating virus.

### Animal-Specific Raw Serum Inhibition of the Influenza NA

**Figure 1:**
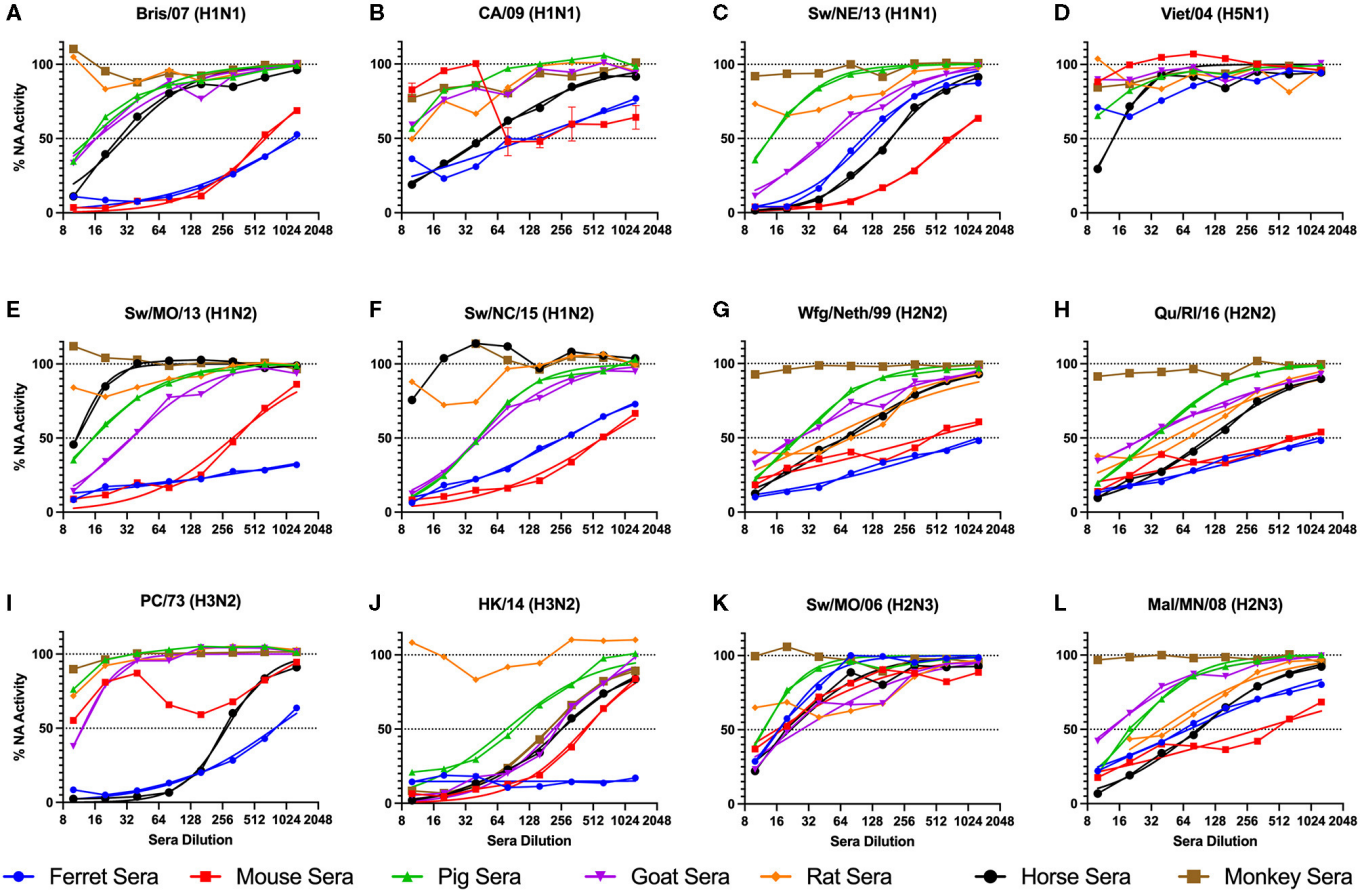
NA inhibition of influenza virus with addition of raw animal sera. A panel of influenza viruses were tested including N1 **(A-D)**, N2 **(E-J)**, and N3 **(K,L)** NA subtypes. The sera were two-fold serially diluted from the reciprocal dilutions of 10-1,280. Non-linear regression was conducted, and the regression that resulted in estimable parameters (as indicated in Table 2) are shown. The NA activity was normalized to 100% of a “virus only with no sera” control.

Sera collected from seven different sources were tested for the ability to inhibit the influenza virus NA activity as tested in the ELLA assay with fetuin substrate (Figure 1). Each serum sample was tested against 12 influenza viruses containing either NA type N1, N2, or N3. There were four swine origin viruses and three avian origin viruses. The 50% NAI titers were estimable for only some virus and serum pairs (Table 2).

**Table 2:** Non-linear regression fits of raw serum inhibition of Type A influenza viruses.

	**Result**	**Ferret**	**Mouse**	**Pig**	**Goat**	**Rat**	**Horse**	**Monkey**
Bris/07	EC_50_	1,251	647.7	14.32	16.33		29.90	
	95% EC_50_	997.3, 1,685	588.3, 717.2	11.79, 16.77	10.84, 21.99		24.86, 36.02	
	Adj. R^2^	0.9493	0.9837	0.9541	0.8703		0.9497	
CA/09	EC_50_	125.4					48.24	
	95% EC_50_	78.76, 200.4					41.95, 55.31	
	Adj. R^2^	0.8110					0.9799	
Sw/NE/13	EC_50_	117.4	725.9	13.89	51.26		189.7	
	95% EC_50_	102.9, 134.3	674.2, 785.2	13.25, 14.53	43.64, 60.15		172.5, 208.8	
	Adj. R^2^	0.9821	0.9917	0.9946	0.9737		0.9903	
Viet/04	EC_50_						14.04	
	95% EC_50_						11.71, 16.51	
	Adj. R^2^						0.8780	
Sw/MO/13	EC_50_	23,011	318.1	15.51	35.70		10.66	
	95% EC_50_	10,183, 75,817	258.1, 392.4	14.72, 16.30	30.74, 41.39		10.04, 11.27	
	Adj. R^2^	0.9040	0.9466	0.9954	0.9721		0.9782	
Sw/NC/15	EC_50_	270.4	636.1	41.73	45.57			
	95% EC_50_	244.0, 300.5	538.2, 770.2	37.61, 46.28	38.96, 53.28			
	Adj. R^2^	0.9891	0.9653	0.9866	0.9723			
Wfg/Neth/99	EC_50_	1,279	425.0	26.01	25.89	47.34	69.02	
	95% EC_50_	997.4, 1,727	265.3, 816.6	24.16, 27.96	18.98, 33.75	30.45, 68.96	62.62, 76.01	
	Adj. R^2^	0.9651	0.8161	0.9920	0.9261	0.8721	0.9907	
Qu/RI/16	EC_50_	1,214	817.3	32.91	27.97	48.04	111.1	
	95% EC_50_	957.5, 1,611	491.6, 1749	29.91, 36.16	23.18, 33.17	34.61, 64.45	96.41, 127.9	
	Adj. R^2^	0.9695	0.8347	0.9889	0.9710	0.9182	0.9804	
PC/73	EC_50_	798.4			12.10		280.0	
	95% EC_50_	666.1, 992.5			10.83, 13.39		262.2, 299.1	
	Adj. R^2^	0.9555			0.9519		0.9936	
HK/14	EC_50_		424.5	78.03	216.6		258.7	194.9
	95% EC_50_		379.5, 475.3	57.20, 104.9	178.9, 261.1		240.4, 278.4	179.7, 211.4
	Adj. R^2^		0.9810	0.9193	0.9618		0.9941	0.9927
Sw/MO/06	EC_50_	16.96	16.00	11.98	27.26		21.67	
	95% EC_50_	15.25, 18.78	10.44, 21.55	10.65, 13.23	16.58, 40.81		15.70, 28.64	
	Adj. R^2^	0.9745	0.8812	0.9534	0.8365		0.8724	
Mal/MN/08	EC_50_	72.27	329.5	21.98	12.77	39.23	87.82	
	95% EC_50_	61.88, 84.12	222.4, 537.1	20.23, 23.84	10.09, 15.37	30.55, 48.54	81.79, 94.29	
	Adj. R^2^	0.9753	0.8585	0.9883	0.9505	0.9505	0.9950	

*The 50% NA inhibitory concentration estimate (EC_50_, half maximal effective concentration), the 95% profile-likelihood confidence intervals, and the adjusted R-squared (Adj. R^2^) were determined for each fit. Sera and virus pairs that resulted in an unstable estimate or did not have an estimate >10 are not shown*.

Ferret sera inhibited ELLA activity by 11 of the 12 viruses with a dilution titer >1:10 and 9 viruses with a titer >1:100 (Table 3). The rat sera inhibited the least number of viral NAs, inhibiting ELLA activity by three of the H2 viruses. Interestingly, not all animal sera inhibited all the same viruses (Table 3). For example, the Bris/07 (H1N1) virus was inhibited by ferret and mouse sera at a dilution >1:100, by pig, goat, and horse sera at a dilution >1:10, and was not inhibited by either rat or monkey sera. This variation was observed for other subtypes and host origin isolates. The Wfg/Neth/99 (H2N2) had a similar inhibition profile. The HK/14 (H3N2) virus was inhibited by the greatest number of sera. There was no distinguishable viral characteristic, such as host origin or HA or NA subtype, that was correlated with pattern of sera inhibition.

**Table 3:** NA inhibition of raw sera stratified by host origin.

**NA**	**HA**	**Host**	**Strain**	**Ferret**	**Mouse**	**Pig**	**Goat**	**Rat**	**Horse**	**Monkey**	**>10**	**>100**
N1	H1	Human	Bris/07	1,251	648	14	16	<10	30	<10	5	2
	H1	Human	CA/09	125	<10	<10	<10	<10	48	<10	2	1
	H1	Swine	Sw/NE/13	117	726	14	51	<10	190	<10	5	3
	H5	Human	Viet/04	<10	<10	<10	<10	<10	14	<10	1	0
N2	H1	Swine	Sw/MO/13	>1,280	318	16	36	<10	11	<10	5	2
	H1	Swine	Sw/NC/15	270	636	42	46	<10	<10	<10	4	2
	H2	Avian	Wfg/Neth/99	>1,280	425	26	26	47	69	<10	6	2
	H2	Avian	Qu/RI/16	1,214	817	33	28	48	111	<10	6	3
	H3	Human	PC/73	798	<10	<10	12	<10	280	<10	3	2
	H3	Human	HK/14	>1,280	425	78	217	<10	259	195	6	5
N3	H2	Swine	Sw/MO/06	17	16	12	27	<10	22	<10	5	0
	H2	Avian	Mal/MN/08	72	330	22	13	39	88	<10	6	1
Number of viruses with NAI > 10				11	9	9	10	3	11	1	54	–
Number of viruses with NAI > 100				9	8	0	1	0	4	1	–	23

*Viruses tested are separated by NA subtype, HA subtype, and host origin. The reciprocal NAI 50% titer for each virus and serum pair is shown from the non-linear regression estimates. The number of viruses or sera with NAI 50% titers >1:10 and 1:100 is tabulated by serum origin and by virus, respectively*.

## Discussion

Influenza vaccine formulations, including live-attenuated virus, whole-inactivated virus, and protein subunit minutes, use NA as a vaccine component to elicit NA-specific antibodies (28). However, components in raw sera have anti-NA properties that result in inhibition of NA activity. The ELLA is used to measure antibody-mediated NA inhibition for cleaving a large substrate, and has been used to assess the effectiveness of NA-containing vaccines and anti-NA antibodies (29–32).

In this study, seven raw animal sera were tested for inhibition of virus in the ELLA assay (Table 2). All sera, regardless of species, inhibited at least one influenza virus (50% inhibition) with a dilution of >1:10. Five of the seven samples inhibited 50% NAI activity at a titer of >1:100. Sera contain innate host influenza inhibitors, such as complement protein of the α-, β-, and γ-class serum inhibitors. In horse and pig sera, the α-2-macroglobulin (γ-class) is one of the major innate influenza virus-neutralizing factors (33, 34). The γ-class inhibitors express sialic acids that bind specifically to the HA protein on influenza viruses and may inhibit the NA through steric interactions. These γ-class inhibitors are inactivated through RDE treatment using *Vibrio cholerae* NA and are resistant to viral sialidase activity (34, 35). There appear to be minor innate factors that result in the ability of horse and pig sera to inhibit different viruses in the panel.

Not all sera inhibited NA activity of all viruses. There were distinct inhibition profiles against specific influenza viruses in the panel. Innate inhibitors interact with influenza viruses through competitive binding of sialic acids to the HA protein receptor-binding site (RBS) (α- and γ-class) and with mannose-binding lectins (β-class) (36, 37). Depending on the host origin of the virus, the HA RBS may have stronger affinity for α-2,3 or α-2,6 sialic acids. The glycosylation of HA proteins has been associated with mannose-binding lectins (37). Further research into the contributions of HA sialic acid binding specificity and the glycosylation of HA and NA surface proteins is needed to determine if it is significantly impacting the variation of NA inhibition observed here across the different viruses.

The innate NA inhibition of different species sera is useful for determining the appropriate treatment before conducting for ELLA assays. To account for the innate inhibitors observed here, sera may either be heat treated or RDE treated overnight at 37°C to cleave competing sialic acids from α- and γ-class inhibitors and heat inactivated at 56°C for a minimum of 30 min to inactivate the heat-labile β-class inhibitors and up to 8 h to fully inactivate the *V. cholerae* NA, when used with ferret sera (20). Immunoglobulins vary in their heat stability with IgG being more stable than IgA which is more stable than IgM (38). With researchers using different inactivation methods, it may be inappropriate to compare titers between sera heat inactivated for 30 min to RDE-treated sera that is heat inactivated for 8 h.

However, one of the major limitations of the study design was the inability to quantify within-species variability due to the limited sources of the sera. This variability can be further investigated to determine if age, sex, or husbandry practices, such as farm or laboratory origin animals, have any effect on the results. Furthermore, the serum inactivation procedure for conducting the ELLA may be different between species. To determine the appropriate method, positive control antiserum is necessary to confirm that no loss in NA-specific antibodies is observed during treatment. Given the wide panel of viruses and different animal models tested here, those samples were not available. Lastly, the wide variability in the NA activity titers observed between viruses (Table 1) may either be from increased enzymatic capacity, i.e., a virus' NA protein cleaves more sialic acid at a higher rate than another viral NA, or from having a higher NA content per PFU. Therefore, why different viruses had such variability in NA activity was undetermined.

In conclusion, with the increase in NA research, the RDE treatment, the inactivation time, and the temperature used to inactivate sialidases should be clearly described with the negative control data provided for each viral strain with serum species used for the assay in order to accurately interpret the results. This information will allow for comparison across species or if comparison of anti-NA serological results need to assessed within the same species.

## Data Availability Statement

The original contributions presented in the study are included in the article/supplementary material, further inquiries can be directed to the corresponding author/s.

## Ethics Statement

The animal study was reviewed and approved by IACUC of University of Georgia.

## Author Contributions

AS: concept, writing, analysis, and statistics. TR: editing and writing. All authors contributed to the article and approved the submitted version.

## Conflict of Interest

The authors declare that the research was conducted in the absence of any commercial or financial relationships that could be construed as a potential conflict of interest.

## Publisher's Note

All claims expressed in this article are solely those of the authors and do not necessarily represent those of their affiliated organizations, or those of the publisher, the editors and the reviewers. Any product that may be evaluated in this article, or claim that may be made by its manufacturer, is not guaranteed or endorsed by the publisher.
